# (1,2-Dicarba-*closo*-dodeca­boran­yl)trimethyl­methanaminium iodide

**DOI:** 10.1107/S160053681102928X

**Published:** 2011-07-30

**Authors:** Jong-Dae Lee, Won-Sik Han, Il-Hwan Suh, Sang Ook Kang

**Affiliations:** a Department of Chemistry, College of Natural Sciences, Chosun University, Gwangju 501-759, Republic of Korea; b Department of Advanced Materials Chemistry, Korea University, Sejong Campus, Chungnam 339-700, Republic of Korea; c ReSEAT Program, Korea Institute of Science and Technology Information, 335 Eoeun-dong, Yuseong-gu, Daejeon 305-806, Republic of Korea

## Abstract

The title compound, [1-(CH_3_)_3_NCH_2_-1,2-C_2_B_10_H_11_]^+^·I^−^ or C_6_H_22_B_10_N^+^·I^−^, was obtained by the reaction of (1,2-dicarba-*closo*-dodeca­boran­yl)dimethyl­methanamine with methyl iodide. The asymmetric unit contains two iodide anions and two (*o*-carboran­yl)tetra­methyl­ammonium cations. The bond lengths and angles in the carborane cage are within normal ranges, but the N—C_methyl­ene_—C_cage_ angle is very large [120.2 (2)°] because of repulsion between the carborane and tetra­methyl­ammonium units. In the crystal, ions are linked through C—H⋯I hydrogen bonds.

## Related literature

For background to quaternaryammonium salts, see: Wiebcke & Felsche (2001 ▶); Zhang *et al.* (2004 ▶); Carr *et al.* (2006 ▶). For background to *o*-carborane structures, see: Davidson *et al.* (1996 ▶); Lee *et al.* (2000 ▶); Welch *et al.* (2001 ▶). For a related structure, see: Lee *et al.* (1999 ▶).
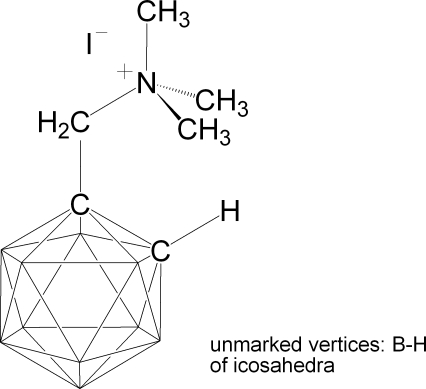


## Experimental

### 

#### Crystal data

C_6_H_22_B_10_N^+^·I^−^
*M* *_r_* = 343.25
Monoclinic, 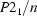

*a* = 6.7435 (14) Å
*b* = 25.013 (5) Å
*c* = 18.694 (4) Å
β = 94.800 (4)°
*V* = 3142.2 (11) Å^3^
*Z* = 8
Mo *K*α radiation
μ = 2.01 mm^−1^
*T* = 293 K
0.28 × 0.25 × 0.20 mm

#### Data collection

Bruker SMART 1000 CCD diffractometer
Absorption correction: multi-scan (*SADABS*; Sheldrick, 2008 ▶) *T*_min_ = 0.603, *T*_max_ = 0.689
32138 measured reflections
7772 independent reflections
5834 reflections with *I* > 2σ(*I*)
*R*_int_ = 0.032

#### Refinement

*R*[*F* ^2^ > 2σ(*F* ^2^)] = 0.031
*wR*(*F* ^2^) = 0.076
*S* = 1.02
7772 reflections
325 parameters
H-atom parameters constrained
Δρ_max_ = 0.88 e Å^−3^
Δρ_min_ = −0.92 e Å^−3^

### 

Data collection: *SMART* (Bruker, 1999 ▶); cell refinement: *SAINT-Plus* (Bruker, 1999 ▶); data reduction: *SAINT-Plus*; program(s) used to solve structure: *SHELXS97* (Sheldrick, 2008 ▶); program(s) used to refine structure: *SHELXL97* (Sheldrick, 2008 ▶); molecular graphics: *ORTEP-3 for Windows* (Farrugia, 1997 ▶); software used to prepare material for publication: *SHELXL97*.

## Supplementary Material

Crystal structure: contains datablock(s) global, I. DOI: 10.1107/S160053681102928X/dn2706sup1.cif

Structure factors: contains datablock(s) I. DOI: 10.1107/S160053681102928X/dn2706Isup2.hkl

Additional supplementary materials:  crystallographic information; 3D view; checkCIF report

## Figures and Tables

**Table 1:** Hydrogen-bond geometry (Å, °)

*D*—H⋯*A*	*D*—H	H⋯*A*	*D*⋯*A*	*D*—H⋯*A*
C2—H2⋯I1	1.10	3.03	3.946 (3)	141
C3—H3*B*⋯I1	0.97	2.94	3.904 (3)	172
C23—H23*B*⋯I1	0.97	2.96	3.921 (3)	170

**Fig. 1.**
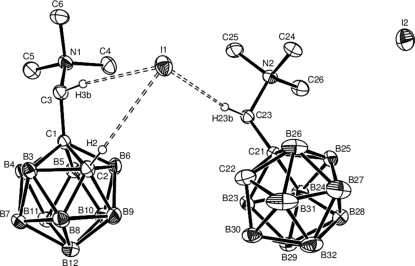
The molecular structure of (I), showing the atom-numbering scheme. Displacement ellipsoids are drawn at the 30% probability level, and H atoms (except for H2, H3B, and H23B) have been omitted for clarity. Dashed lines indicate intra- and intermolecular hydrogen bonds.

## supplementary crystallographic information

### Comment

*N*,*N*-dimethyl-(1,2-dicarba-*closo*-dodecaboranyl)methanamine is a useful intramolecular coordinating ligand for many different metals (Lee *et al.*, 1999, 2000). Since the starting material is an oil, it could not be characterized by X-ray diffraction. However, we found that the corresponding methyl iodide forms crystals suitable for crystallographic study. We report here the synthesis and structure of the title compound (I).

In (I), shown in Fig. 1 and Table 1, the average bond length N—C [1.508 (2) Å] in the tetramethylammonium unit is similar to 1.492 (5) Å in an *o*-carboranyl organogallium compound [Cl_3_Ga][N(CH_3_)_2_CH_2_-1,2-C_2_B_10_H_11_] (Lee *et al.*, 1999), 1.505 (2) Å in a benzyltrimethylammonium hydroxide trihydrate system (Wiebcke & Felsche, 2001) and 1.478 (5) Å in a tetramethylammonium pentaborate 0.25-hydrate compound (Zhang *et al.*, 2004).

The average bond angle of C_cage_—C_methylene_—N [120.3 (1)°] in (I) is almost same as 120.5 (6)° of *o*-carboranyl organogallium compound (Lee *et al.*, 1999). Their large difference from the tetrahedral angle might be attributable to the repulsion between the carborane and tetramethylammonium unit. On the other hand, Carr and co-workers reported far smaller angle [113.5 (2)°] for the same methylene unit of [H_3_NCH_2_C_2_B_10_H_11_][H_3_CCH_2_CB_11_H_11_] with a smaller methylammonium unit than tetramethylammonium one. Compared with these two values, it would seem the above-mentioned repulsion logic would be affirmatively accepted.

The carborane moiety forms an icosahedrons consisting of twenty triangles with sides of the average bond length of C_cage_—C_cage_ 1.659 (3), C_cage_—B_cage_ 1.713 (1), and B_cage_—B_cage_ 1.771 (1) Å. All of the compounds containing mono-substituted *o*-carboranes (Lee *et al.*, 1999; Welch, *et al.*, 2001) including unsubstituted *ortho*-, *meta*-, and *para*-carboranes with hexamethylphosphoramide (Davidson, *et al.*, 1996) surveyed by our group so far exhibit the same trend of d(C_cage_—C_cage_) < d(C_cage_—B_cage_) < d(B_cage_—B_cage_). Since the boron atom has one valence electron less than carbon, this result confirms that the bond lengths will become shorter when more electrons participate in bond formation.

As shown in Table 1 and Figure 1, I1 atom participates in two intramolecular and an intermolecular hydrogen bonds, while I2 atom has only weak interaction with the closest interatomic distances I2···H3A^i^ 3.126 Å and I2···H5A^ii^ 3.126 Å [symmetry code: (i) 0.5 - *x*, 1/2 + *y*, 1.5 - *z*; (ii) -1/2 + *x*, 1.5 - *y*, 1/2 + *z*]. The shortest interdimer's distance B26···H24^iii^ 2.912 Å [symmetry code: (iii) -1 + *x*, *y*, *z*] suggests the dimer's packing of (I) is governed by van der Waals forces.

### Experimental

A mixture of *N*,*N*-dimethyl-(1,2-dicarba-*closo*-dodecaboranyl)methanamine (0.2 g, 1.0 mmol) and 1.2 equiv of methyl iodide (0.17 g, 1.2 mmol) was dissolved in distilled diethyl ether (10 ml) and stirred at room temperature for 30 min. The resulting white solid was collected by filtration and washed with cold diethyl ether (quantitative yield, m.p. 389 K). The purity was checked by ^1^H, ^13^C, and ^11^B NMR spectroscopies. Suitable single crystals of (I) were obtained by recrystallization from acetone.

### Refinement

All of the hydrogen atoms were placed at idealized positions and treated using a riding model, with constrained distances and *U*_iso_(H) values fixed at *xU*_eq_ (parent atom) [C/B—H = 1.1 Å and *x* = 1.2 for carborane H atoms, C—H = 0.97 Å, and *x* = 1.2 for methylene H atoms, and C—H = 0.96 Å and *x* = 1.5 for methyl H atoms].

### Crystal data

**Table:**

C_6_H_22_B_10_N^+^·I^−^	*F*(000) = 1344
*M**_r_* = 343.25	*D*_x_ = 1.451 Mg m^−^^3^
Monoclinic, *P*2_1_/*n*	Mo *K*α radiation, λ = 0.71073 Å
Hall symbol: -P 2yn	Cell parameters from 5251 reflections
*a* = 6.7435 (14) Å	θ = 2.3–27.3°
*b* = 25.013 (5) Å	µ = 2.01 mm^−^^1^
*c* = 18.694 (4) Å	*T* = 293 K
β = 94.800 (4)°	Block, colourless
*V* = 3142.2 (11) Å^3^	0.28 × 0.25 × 0.20 mm
*Z* = 8	

### Data collection

**Table:**

Bruker SMART 1000 CCD diffractometer	7772 independent reflections
Radiation source: fine-focus sealed tube	5834 reflections with *I* > 2σ(*I*)
graphite	*R*_int_ = 0.032
ω scans	θ_max_ = 28.3°, θ_min_ = 1.4°
Absorption correction: multi-scan (*SADABS*; Sheldrick, 2008)	*h* = −8→8
*T*_min_ = 0.603, *T*_max_ = 0.689	*k* = −33→33
32138 measured reflections	*l* = −24→24

### Refinement

**Table:**

Refinement on *F*^2^	Primary atom site location: structure-invariant direct methods
Least-squares matrix: full	Secondary atom site location: difference Fourier map
*R*[*F*^2^ > 2σ(*F*^2^)] = 0.031	Hydrogen site location: inferred from neighbouring sites
*wR*(*F*^2^) = 0.076	H-atom parameters constrained
*S* = 1.02	*w* = 1/[σ^2^(*F*_o_^2^) + (0.0267*P*)^2^ + 2.9488*P*] where *P* = (*F*_o_^2^ + 2*F*_c_^2^)/3
7772 reflections	(Δ/σ)_max_ = 0.002
325 parameters	Δρ_max_ = 0.88 e Å^−^^3^
0 restraints	Δρ_min_ = −0.92 e Å^−^^3^

### Special details

**Table:**

Geometry. All e.s.d.'s (except the e.s.d. in the dihedral angle between two l.s. planes) are estimated using the full covariance matrix. The cell e.s.d.'s are taken into account individually in the estimation of e.s.d.'s in distances, angles and torsion angles; correlations between e.s.d.'s in cell parameters are only used when they are defined by crystal symmetry. An approximate (isotropic) treatment of cell e.s.d.'s is used for estimating e.s.d.'s involving l.s. planes.
Refinement. Refinement of *F*^2^ against ALL reflections. The weighted *R*-factor *wR* and goodness of fit *S* are based on *F*^2^, conventional *R*-factors *R* are based on *F*, with *F* set to zero for negative *F*^2^. The threshold expression of *F*^2^ > σ(*F*^2^) is used only for calculating *R*-factors(gt) *etc*. and is not relevant to the choice of reflections for refinement. *R*-factors based on *F*^2^ are statistically about twice as large as those based on *F*, and *R*- factors based on ALL data will be even larger.

### Fractional atomic coordinates and isotropic or equivalent isotropic displacement parameters (Å^2^)

**Table:**

	*x*	*y*	*z*	*U*_iso_*/*U*_eq_	
I2	0.30391 (3)	0.913655 (9)	0.909132 (12)	0.05540 (7)	
I1	0.26909 (3)	0.657927 (8)	0.774753 (12)	0.04975 (7)	
N1	0.6119 (4)	0.55189 (9)	0.62825 (12)	0.0392 (5)	
B3	0.5023 (5)	0.46207 (12)	0.79957 (17)	0.0363 (6)	
H3	0.3559	0.4472	0.7779	0.044*	
B4	0.7252 (5)	0.44574 (12)	0.76026 (17)	0.0356 (6)	
H4	0.7240	0.4198	0.7127	0.043*	
B5	0.8919 (5)	0.50059 (13)	0.77262 (18)	0.0385 (7)	
H5	0.9993	0.5100	0.7333	0.046*	
B6	0.7713 (5)	0.55209 (13)	0.81841 (17)	0.0406 (7)	
H6	0.7968	0.5948	0.8088	0.049*	
B7	0.6790 (5)	0.42083 (12)	0.84662 (17)	0.0404 (7)	
H7	0.6493	0.3783	0.8559	0.048*	
B8	0.5593 (5)	0.47206 (13)	0.89249 (18)	0.0431 (7)	
H8	0.4497	0.4635	0.9313	0.052*	
B9	0.7252 (6)	0.52692 (14)	0.90438 (18)	0.0462 (8)	
H9	0.7227	0.5536	0.9511	0.055*	
B10	0.9492 (6)	0.51054 (15)	0.86573 (19)	0.0490 (8)	
H10	1.0944	0.5262	0.8874	0.059*	
B11	0.9206 (5)	0.44426 (14)	0.82976 (19)	0.0449 (8)	
H11	1.0474	0.4168	0.8281	0.054*	
B12	0.8182 (6)	0.46069 (14)	0.91206 (18)	0.0480 (8)	
H12	0.8790	0.4440	0.9638	0.058*	
C1	0.6407 (4)	0.50998 (9)	0.75779 (13)	0.0307 (5)	
C2	0.5473 (4)	0.52448 (10)	0.83484 (14)	0.0373 (6)	
H2	0.4198	0.5518	0.8356	0.045*	
C3	0.5144 (4)	0.53300 (10)	0.69337 (14)	0.0353 (6)	
H3A	0.4176	0.5060	0.6774	0.042*	
H3B	0.4406	0.5630	0.7106	0.042*	
C4	0.7509 (5)	0.59801 (12)	0.64407 (18)	0.0549 (8)	
H4A	0.7942	0.6116	0.5999	0.082*	
H4B	0.8643	0.5862	0.6744	0.082*	
H4C	0.6833	0.6257	0.6679	0.082*	
C5	0.7167 (5)	0.50697 (12)	0.59283 (16)	0.0512 (8)	
H5A	0.7591	0.5191	0.5478	0.077*	
H5B	0.6272	0.4773	0.5846	0.077*	
H5C	0.8305	0.4959	0.6235	0.077*	
C6	0.4426 (5)	0.57103 (14)	0.57671 (17)	0.0558 (8)	
H6A	0.3784	0.6009	0.5973	0.084*	
H6B	0.3483	0.5426	0.5676	0.084*	
H6C	0.4935	0.5818	0.5325	0.084*	
N2	0.7834 (3)	0.77280 (8)	0.79247 (11)	0.0325 (5)	
B23	0.8766 (5)	0.67804 (12)	0.96808 (17)	0.0367 (7)	
H23	0.9569	0.6459	0.9426	0.044*	
B24	0.9750 (5)	0.74312 (13)	0.97941 (17)	0.0380 (7)	
H24	1.1216	0.7534	0.9615	0.046*	
B25	0.7765 (6)	0.78988 (13)	0.97298 (18)	0.0479 (8)	
H25	0.7942	0.8304	0.9514	0.058*	
B26	0.5520 (5)	0.75335 (18)	0.9567 (2)	0.0550 (10)	
H26	0.4227	0.7695	0.9239	0.066*	
B27	0.6239 (8)	0.7764 (2)	1.0435 (2)	0.0782 (16)	
H27	0.5411	0.8084	1.0682	0.094*	
B28	0.8867 (7)	0.76998 (15)	1.05814 (19)	0.0594 (11)	
H28	0.9769	0.7979	1.0927	0.071*	
B29	0.9465 (5)	0.70112 (13)	1.05485 (17)	0.0389 (7)	
H29	1.0750	0.6841	1.0872	0.047*	
B30	0.7242 (5)	0.66515 (15)	1.03832 (18)	0.0453 (8)	
H30	0.7053	0.6244	1.0587	0.054*	
B31	0.5269 (6)	0.7114 (2)	1.0320 (2)	0.0712 (14)	
H31	0.3797	0.7005	1.0488	0.085*	
B32	0.7311 (6)	0.72198 (17)	1.09454 (19)	0.0555 (10)	
H32	0.7188	0.7188	1.1527	0.067*	
C21	0.7687 (3)	0.73285 (9)	0.92250 (13)	0.0281 (5)	
C22	0.6242 (4)	0.68780 (13)	0.95729 (16)	0.0488 (8)	
H22	0.5352	0.6600	0.9221	0.059*	
C23	0.7767 (4)	0.72499 (9)	0.84162 (13)	0.0309 (5)	
H23A	0.8930	0.7033	0.8348	0.037*	
H23B	0.6613	0.7039	0.8248	0.037*	
C24	0.6028 (4)	0.80804 (11)	0.79378 (16)	0.0439 (7)	
H24A	0.4848	0.7869	0.7839	0.066*	
H24B	0.6074	0.8355	0.7580	0.066*	
H24C	0.6009	0.8242	0.8403	0.066*	
C25	0.7839 (5)	0.74947 (13)	0.71782 (15)	0.0486 (7)	
H25A	0.6646	0.7291	0.7070	0.073*	
H25B	0.8977	0.7266	0.7156	0.073*	
H25C	0.7899	0.7779	0.6836	0.073*	
C26	0.9719 (4)	0.80439 (12)	0.80762 (16)	0.0450 (7)	
H26A	0.9709	0.8345	0.7757	0.067*	
H26B	1.0843	0.7821	0.8004	0.067*	
H26C	0.9808	0.8168	0.8564	0.067*	

### Atomic displacement parameters (Å^2^)

**Table:**

	*U*^11^	*U*^22^	*U*^33^	*U*^12^	*U*^13^	*U*^23^
I2	0.05902 (14)	0.04948 (13)	0.05873 (14)	0.00517 (10)	0.01109 (10)	−0.00694 (10)
I1	0.04172 (11)	0.04153 (11)	0.06601 (14)	0.00482 (8)	0.00458 (9)	−0.00515 (9)
N1	0.0525 (14)	0.0311 (11)	0.0343 (12)	−0.0003 (10)	0.0058 (10)	0.0047 (9)
B3	0.0390 (16)	0.0297 (14)	0.0411 (17)	−0.0052 (12)	0.0085 (13)	0.0027 (12)
B4	0.0421 (17)	0.0274 (14)	0.0384 (16)	0.0028 (12)	0.0097 (13)	−0.0001 (12)
B5	0.0339 (16)	0.0387 (16)	0.0433 (17)	−0.0013 (13)	0.0058 (13)	0.0018 (13)
B6	0.0488 (19)	0.0333 (15)	0.0399 (17)	−0.0108 (13)	0.0057 (14)	−0.0052 (13)
B7	0.0510 (19)	0.0300 (15)	0.0406 (17)	−0.0011 (13)	0.0054 (14)	0.0077 (13)
B8	0.054 (2)	0.0395 (17)	0.0371 (17)	−0.0033 (14)	0.0116 (15)	0.0043 (13)
B9	0.065 (2)	0.0397 (17)	0.0338 (17)	−0.0077 (16)	0.0063 (15)	−0.0031 (13)
B10	0.048 (2)	0.052 (2)	0.0456 (19)	−0.0099 (16)	−0.0047 (15)	0.0051 (16)
B11	0.0425 (18)	0.0448 (18)	0.0473 (19)	0.0059 (14)	0.0033 (15)	0.0075 (15)
B12	0.060 (2)	0.0464 (19)	0.0371 (18)	−0.0033 (16)	−0.0004 (15)	0.0065 (14)
C1	0.0362 (13)	0.0259 (12)	0.0307 (13)	−0.0020 (10)	0.0070 (10)	−0.0003 (10)
C2	0.0451 (15)	0.0326 (13)	0.0357 (14)	0.0003 (11)	0.0122 (12)	−0.0015 (11)
C3	0.0398 (14)	0.0322 (13)	0.0344 (14)	0.0025 (11)	0.0058 (11)	0.0013 (10)
C4	0.071 (2)	0.0380 (16)	0.0564 (19)	−0.0159 (15)	0.0067 (16)	0.0079 (14)
C5	0.071 (2)	0.0469 (17)	0.0379 (16)	0.0065 (15)	0.0173 (15)	−0.0019 (13)
C6	0.074 (2)	0.0516 (18)	0.0401 (17)	0.0082 (16)	−0.0035 (15)	0.0089 (14)
N2	0.0334 (11)	0.0323 (11)	0.0321 (11)	0.0008 (9)	0.0039 (9)	0.0047 (9)
B23	0.0446 (17)	0.0276 (14)	0.0379 (16)	0.0026 (12)	0.0026 (13)	0.0054 (12)
B24	0.0312 (15)	0.0415 (17)	0.0402 (17)	−0.0075 (12)	−0.0050 (12)	0.0089 (13)
B25	0.072 (2)	0.0325 (16)	0.0386 (18)	0.0116 (16)	0.0014 (16)	−0.0033 (13)
B26	0.0347 (17)	0.088 (3)	0.0447 (19)	0.0240 (18)	0.0151 (15)	0.0210 (19)
B27	0.103 (4)	0.092 (3)	0.043 (2)	0.061 (3)	0.026 (2)	0.007 (2)
B28	0.097 (3)	0.0415 (19)	0.0372 (19)	0.001 (2)	−0.0105 (19)	−0.0030 (15)
B29	0.0345 (16)	0.0450 (18)	0.0363 (16)	−0.0003 (13)	−0.0032 (13)	0.0074 (13)
B30	0.0448 (18)	0.053 (2)	0.0371 (17)	−0.0124 (15)	−0.0004 (14)	0.0158 (15)
B31	0.0376 (19)	0.126 (4)	0.053 (2)	0.019 (2)	0.0188 (17)	0.039 (2)
B32	0.063 (2)	0.073 (3)	0.0321 (17)	0.020 (2)	0.0106 (16)	0.0080 (17)
C21	0.0240 (11)	0.0290 (12)	0.0314 (12)	−0.0008 (9)	0.0026 (9)	0.0026 (10)
C22	0.0415 (16)	0.065 (2)	0.0392 (16)	−0.0214 (14)	−0.0028 (12)	0.0161 (14)
C23	0.0347 (13)	0.0274 (12)	0.0307 (13)	−0.0005 (10)	0.0035 (10)	0.0020 (10)
C24	0.0394 (15)	0.0400 (15)	0.0521 (17)	0.0115 (12)	0.0030 (13)	0.0123 (13)
C25	0.067 (2)	0.0497 (17)	0.0295 (14)	0.0054 (15)	0.0049 (13)	0.0047 (12)
C26	0.0378 (15)	0.0455 (16)	0.0516 (17)	−0.0081 (12)	0.0034 (13)	0.0124 (13)

### Geometric parameters (Å, °)

**Table:**

N1—C4	1.500 (4)	N2—C26	1.503 (3)
N1—C3	1.507 (3)	N2—C24	1.505 (3)
N1—C5	1.510 (4)	N2—C23	1.511 (3)
N1—C6	1.509 (4)	N2—C25	1.513 (3)
B3—C2	1.712 (4)	B23—C22	1.714 (4)
B3—C1	1.743 (4)	B23—C21	1.741 (4)
B3—B7	1.756 (5)	B23—B29	1.749 (4)
B3—B8	1.765 (5)	B23—B30	1.763 (5)
B3—B4	1.775 (4)	B23—B24	1.764 (4)
B3—H3	1.1000	B23—H23	1.1000
B4—C1	1.704 (4)	B24—C21	1.699 (4)
B4—B11	1.772 (5)	B24—B28	1.766 (5)
B4—B5	1.776 (4)	B24—B25	1.774 (5)
B4—B7	1.782 (4)	B24—B29	1.782 (4)
B4—H4	1.1000	B24—H24	1.1000
B5—C1	1.710 (4)	B25—C21	1.709 (4)
B5—B10	1.769 (5)	B25—B26	1.772 (6)
B5—B11	1.769 (5)	B25—B28	1.771 (5)
B5—B6	1.780 (5)	B25—B27	1.772 (6)
B5—H5	1.1000	B25—H25	1.1000
B6—C2	1.712 (4)	B26—C22	1.710 (5)
B6—C1	1.733 (4)	B26—C21	1.722 (4)
B6—B10	1.767 (5)	B26—B27	1.751 (6)
B6—B9	1.778 (5)	B26—B31	1.774 (5)
B6—H6	1.1000	B26—H26	1.1000
B7—B8	1.773 (5)	B27—B31	1.757 (8)
B7—B12	1.783 (5)	B27—B32	1.779 (6)
B7—B11	1.784 (5)	B27—B28	1.778 (7)
B7—H7	1.1000	B27—H27	1.1000
B8—C2	1.695 (4)	B28—B32	1.768 (6)
B8—B9	1.773 (5)	B28—B29	1.771 (5)
B8—B12	1.776 (5)	B28—H28	1.1000
B8—H8	1.1000	B29—B30	1.753 (5)
B9—C2	1.695 (5)	B29—B32	1.765 (5)
B9—B12	1.773 (5)	B29—H29	1.1000
B9—B10	1.776 (5)	B30—C22	1.703 (4)
B9—H9	1.1000	B30—B31	1.761 (6)
B10—B11	1.794 (5)	B30—B32	1.766 (6)
B10—B12	1.793 (5)	B30—H30	1.1000
B10—H10	1.1000	B31—C22	1.699 (5)
B11—B12	1.786 (5)	B31—B32	1.750 (6)
B11—H11	1.1000	B31—H31	1.1000
B12—H12	1.1000	B32—H32	1.1000
C1—C3	1.528 (4)	C21—C23	1.530 (3)
C1—C2	1.660 (3)	C21—C22	1.658 (4)
C2—H2	1.1000	C22—H22	1.1000
C3—H3A	0.9700	C23—H23A	0.9700
C3—H3B	0.9700	C23—H23B	0.9700
C4—H4A	0.9600	C24—H24A	0.9600
C4—H4B	0.9600	C24—H24B	0.9600
C4—H4C	0.9600	C24—H24C	0.9600
C5—H5A	0.9600	C25—H25A	0.9600
C5—H5B	0.9600	C25—H25B	0.9600
C5—H5C	0.9600	C25—H25C	0.9600
C6—H6A	0.9600	C26—H26A	0.9600
C6—H6B	0.9600	C26—H26B	0.9600
C6—H6C	0.9600	C26—H26C	0.9600
			
C4—N1—C3	112.9 (2)	C26—N2—C24	111.2 (2)
C4—N1—C5	110.5 (2)	C26—N2—C23	111.7 (2)
C3—N1—C5	111.9 (2)	C24—N2—C23	112.83 (19)
C4—N1—C6	108.0 (2)	C26—N2—C25	108.0 (2)
C3—N1—C6	104.9 (2)	C24—N2—C25	107.8 (2)
C5—N1—C6	108.2 (2)	C23—N2—C25	105.0 (2)
C2—B3—C1	57.41 (15)	C22—B23—C21	57.34 (16)
C2—B3—B7	104.6 (2)	C22—B23—B29	104.5 (2)
C1—B3—B7	105.2 (2)	C21—B23—B29	105.3 (2)
C2—B3—B8	58.33 (17)	C22—B23—B30	58.61 (18)
C1—B3—B8	105.3 (2)	C21—B23—B30	105.2 (2)
B7—B3—B8	60.48 (19)	B29—B23—B30	59.89 (18)
C2—B3—B4	103.9 (2)	C22—B23—B24	104.0 (2)
C1—B3—B4	57.94 (15)	C21—B23—B24	57.98 (15)
B7—B3—B4	60.62 (18)	B29—B23—B24	60.96 (18)
B8—B3—B4	108.5 (2)	B30—B23—B24	108.4 (2)
C2—B3—H3	125.1	C22—B23—H23	125.0
C1—B3—H3	124.4	C21—B23—H23	124.4
B7—B3—H3	122.5	B29—B23—H23	122.6
B8—B3—H3	121.7	B30—B23—H23	121.8
B4—B3—H3	122.3	B24—B23—H23	122.2
C1—B4—B3	60.09 (16)	C21—B24—B23	60.33 (16)
C1—B4—B11	105.4 (2)	C21—B24—B28	105.3 (2)
B3—B4—B11	107.7 (2)	B23—B24—B28	107.5 (2)
C1—B4—B5	58.79 (16)	C21—B24—B25	58.90 (17)
B3—B4—B5	108.5 (2)	B23—B24—B25	109.0 (2)
B11—B4—B5	59.82 (18)	B28—B24—B25	60.0 (2)
C1—B4—B7	105.7 (2)	C21—B24—B29	105.6 (2)
B3—B4—B7	59.15 (18)	B23—B24—B29	59.10 (17)
B11—B4—B7	60.25 (19)	B28—B24—B29	59.90 (19)
B5—B4—B7	108.0 (2)	B25—B24—B29	108.2 (2)
C1—B4—H4	123.7	C21—B24—H24	123.7
B3—B4—H4	121.4	B23—B24—H24	121.2
B11—B4—H4	122.4	B28—B24—H24	122.6
B5—B4—H4	121.5	B25—B24—H24	121.1
B7—B4—H4	122.4	B29—B24—H24	122.5
C1—B5—B10	105.8 (2)	C21—B25—B26	59.24 (19)
C1—B5—B11	105.3 (2)	C21—B25—B28	104.7 (2)
B10—B5—B11	60.9 (2)	B26—B25—B28	107.3 (3)
C1—B5—B4	58.50 (16)	C21—B25—B24	58.36 (16)
B10—B5—B4	108.7 (2)	B26—B25—B24	107.5 (2)
B11—B5—B4	59.96 (18)	B28—B25—B24	59.8 (2)
C1—B5—B6	59.51 (17)	C21—B25—B27	105.0 (3)
B10—B5—B6	59.7 (2)	B26—B25—B27	59.2 (3)
B11—B5—B6	108.5 (2)	B28—B25—B27	60.2 (2)
B4—B5—B6	108.2 (2)	B24—B25—B27	107.8 (2)
C1—B5—H5	124.2	C21—B25—H25	124.4
B10—B5—H5	121.7	B26—B25—H25	121.9
B11—B5—H5	121.9	B28—B25—H25	122.5
B4—B5—H5	121.5	B24—B25—H25	121.8
B6—B5—H5	121.3	B27—B25—H25	122.4
C2—B6—C1	57.59 (15)	C22—B26—C21	57.77 (17)
C2—B6—B10	104.2 (2)	C22—B26—B27	104.6 (3)
C1—B6—B10	104.9 (2)	C21—B26—B27	105.3 (3)
C2—B6—B9	58.07 (18)	C22—B26—B25	104.8 (2)
C1—B6—B9	105.0 (2)	C21—B26—B25	58.53 (17)
B10—B6—B9	60.1 (2)	B27—B26—B25	60.4 (3)
C2—B6—B5	103.9 (2)	C22—B26—B31	58.3 (2)
C1—B6—B5	58.22 (16)	C21—B26—B31	105.0 (2)
B10—B6—B5	59.8 (2)	B27—B26—B31	59.8 (3)
B9—B6—B5	107.5 (2)	B25—B26—B31	107.8 (3)
C2—B6—H6	125.0	C22—B26—H26	124.5
C1—B6—H6	124.2	C21—B26—H26	124.0
B10—B6—H6	123.0	B27—B26—H26	122.8
B9—B6—H6	122.2	B25—B26—H26	122.2
B5—B6—H6	122.8	B31—B26—H26	122.3
B3—B7—B8	60.02 (18)	B26—B27—B31	60.8 (3)
B3—B7—B12	108.1 (2)	B26—B27—B25	60.4 (2)
B8—B7—B12	59.9 (2)	B31—B27—B25	108.6 (3)
B3—B7—B11	108.1 (2)	B26—B27—B32	108.3 (3)
B8—B7—B11	107.9 (2)	B31—B27—B32	59.3 (2)
B12—B7—B11	60.1 (2)	B25—B27—B32	108.0 (3)
B3—B7—B4	60.23 (17)	B26—B27—B28	107.9 (3)
B8—B7—B4	107.9 (2)	B31—B27—B28	107.0 (3)
B12—B7—B4	107.7 (2)	B25—B27—B28	59.9 (2)
B11—B7—B4	59.58 (18)	B32—B27—B28	59.6 (2)
B3—B7—H7	121.5	B26—B27—H27	121.2
B8—B7—H7	121.8	B31—B27—H27	121.8
B12—B7—H7	121.7	B25—B27—H27	121.3
B11—B7—H7	121.8	B32—B27—H27	122.0
B4—B7—H7	121.9	B28—B27—H27	122.4
C2—B8—B3	59.26 (17)	B24—B28—B32	108.4 (3)
C2—B8—B9	58.46 (18)	B24—B28—B25	60.21 (19)
B3—B8—B9	108.4 (2)	B32—B28—B25	108.6 (3)
C2—B8—B7	104.5 (2)	B24—B28—B29	60.50 (19)
B3—B8—B7	59.50 (18)	B32—B28—B29	59.8 (2)
B9—B8—B7	108.2 (2)	B25—B28—B29	108.8 (2)
C2—B8—B12	104.4 (2)	B24—B28—B27	107.9 (3)
B3—B8—B12	108.0 (2)	B32—B28—B27	60.2 (3)
B9—B8—B12	59.9 (2)	B25—B28—B27	59.9 (2)
B7—B8—B12	60.3 (2)	B29—B28—B27	108.0 (3)
C2—B8—H8	124.9	B24—B28—H28	121.5
B3—B8—H8	121.3	B32—B28—H28	121.5
B9—B8—H8	121.3	B25—B28—H28	121.3
B7—B8—H8	122.4	B29—B28—H28	121.5
B12—B8—H8	122.4	B27—B28—H28	121.9
C2—B9—B8	58.48 (18)	B23—B29—B30	60.46 (19)
C2—B9—B12	104.6 (2)	B23—B29—B32	108.7 (2)
B8—B9—B12	60.1 (2)	B30—B29—B32	60.3 (2)
C2—B9—B10	104.5 (2)	B23—B29—B28	108.0 (2)
B8—B9—B10	108.6 (2)	B30—B29—B28	108.2 (3)
B12—B9—B10	60.7 (2)	B32—B29—B28	60.0 (2)
C2—B9—B6	59.03 (18)	B23—B29—B24	59.94 (17)
B8—B9—B6	108.5 (2)	B30—B29—B24	108.1 (2)
B12—B9—B6	108.5 (2)	B32—B29—B24	107.8 (2)
B10—B9—B6	59.65 (19)	B28—B29—B24	59.6 (2)
C2—B9—H9	125.0	B23—B29—H29	121.4
B8—B9—H9	121.2	B30—B29—H29	121.4
B12—B9—H9	122.1	B32—B29—H29	121.5
B10—B9—H9	122.2	B28—B29—H29	121.9
B6—B9—H9	121.2	B24—B29—H29	122.0
B6—B10—B5	60.46 (18)	C22—B30—B29	104.8 (2)
B6—B10—B9	60.2 (2)	C22—B30—B23	59.26 (18)
B5—B10—B9	108.0 (2)	B29—B30—B23	59.65 (18)
B6—B10—B11	108.0 (2)	C22—B30—B31	58.7 (2)
B5—B10—B11	59.54 (19)	B29—B30—B31	107.7 (3)
B9—B10—B11	107.3 (2)	B23—B30—B31	108.3 (2)
B6—B10—B12	108.0 (3)	C22—B30—B32	104.5 (3)
B5—B10—B12	107.5 (2)	B29—B30—B32	60.2 (2)
B9—B10—B12	59.6 (2)	B23—B30—B32	107.9 (2)
B11—B10—B12	59.7 (2)	B31—B30—B32	59.5 (2)
B6—B10—H10	121.3	C22—B30—H30	124.6
B5—B10—H10	121.8	B29—B30—H30	122.5
B9—B10—H10	122.0	B23—B30—H30	121.3
B11—B10—H10	122.2	B31—B30—H30	121.6
B12—B10—H10	122.0	B32—B30—H30	122.6
B5—B11—B4	60.22 (18)	C22—B31—B32	105.4 (2)
B5—B11—B7	108.2 (2)	C22—B31—B27	104.9 (3)
B4—B11—B7	60.16 (18)	B32—B31—B27	61.0 (3)
B5—B11—B12	107.8 (2)	C22—B31—B30	58.9 (2)
B4—B11—B12	108.0 (2)	B32—B31—B30	60.4 (2)
B7—B11—B12	59.93 (19)	B27—B31—B30	109.1 (3)
B5—B11—B10	59.54 (19)	C22—B31—B26	59.0 (2)
B4—B11—B10	107.8 (2)	B32—B31—B26	108.6 (3)
B7—B11—B10	108.0 (2)	B27—B31—B26	59.5 (2)
B12—B11—B10	60.1 (2)	B30—B31—B26	108.6 (2)
B5—B11—H11	121.8	C22—B31—H31	124.6
B4—B11—H11	121.6	B32—B31—H31	121.7
B7—B11—H11	121.6	B27—B31—H31	122.0
B12—B11—H11	121.7	B30—B31—H31	120.8
B10—B11—H11	121.9	B26—B31—H31	121.4
B9—B12—B8	59.9 (2)	B31—B32—B29	107.7 (3)
B9—B12—B7	107.7 (2)	B31—B32—B30	60.1 (2)
B8—B12—B7	59.76 (19)	B29—B32—B30	59.54 (19)
B9—B12—B11	107.8 (2)	B31—B32—B28	107.7 (3)
B8—B12—B11	107.7 (2)	B29—B32—B28	60.2 (2)
B7—B12—B11	59.96 (19)	B30—B32—B28	107.7 (2)
B9—B12—B10	59.8 (2)	B31—B32—B27	59.7 (3)
B8—B12—B10	107.7 (2)	B29—B32—B27	108.2 (3)
B7—B12—B10	108.0 (2)	B30—B32—B27	107.9 (3)
B11—B12—B10	60.2 (2)	B28—B32—B27	60.1 (3)
B9—B12—H12	121.9	B31—B32—H32	121.9
B8—B12—H12	122.0	B29—B32—H32	121.8
B7—B12—H12	121.8	B30—B32—H32	121.9
B11—B12—H12	121.8	B28—B32—H32	121.7
B10—B12—H12	121.7	B27—B32—H32	121.6
C3—C1—C2	112.0 (2)	C23—C21—C22	111.8 (2)
C3—C1—B4	122.7 (2)	C23—C21—B24	122.9 (2)
C2—C1—B4	109.45 (19)	C22—C21—B24	109.54 (19)
C3—C1—B5	131.2 (2)	C23—C21—B25	130.6 (2)
C2—C1—B5	109.4 (2)	C22—C21—B25	110.0 (2)
B4—C1—B5	62.71 (17)	B24—C21—B25	62.74 (19)
C3—C1—B6	120.4 (2)	C23—C21—B26	120.5 (2)
C2—C1—B6	60.57 (16)	C22—C21—B26	60.8 (2)
B4—C1—B6	113.9 (2)	B24—C21—B26	113.5 (2)
B5—C1—B6	62.28 (18)	B25—C21—B26	62.2 (2)
C3—C1—B3	109.2 (2)	C23—C21—B23	109.54 (19)
C2—C1—B3	60.35 (16)	C22—C21—B23	60.52 (17)
B4—C1—B3	61.97 (16)	B24—C21—B23	61.69 (17)
B5—C1—B3	113.2 (2)	B25—C21—B23	113.2 (2)
B6—C1—B3	112.8 (2)	B26—C21—B23	112.7 (2)
C1—C2—B9	112.2 (2)	C21—C22—B30	111.9 (2)
C1—C2—B8	112.4 (2)	C21—C22—B31	111.5 (3)
B9—C2—B8	63.06 (19)	B30—C22—B31	62.3 (2)
C1—C2—B6	61.84 (16)	C21—C22—B26	61.45 (18)
B9—C2—B6	62.90 (19)	B30—C22—B26	114.6 (3)
B8—C2—B6	115.5 (2)	B31—C22—B26	62.7 (2)
C1—C2—B3	62.24 (16)	C21—C22—B23	62.14 (16)
B9—C2—B3	114.8 (2)	B30—C22—B23	62.13 (19)
B8—C2—B3	62.41 (18)	B31—C22—B23	113.6 (2)
B6—C2—B3	115.4 (2)	B26—C22—B23	114.6 (2)
C1—C2—H2	120.0	C21—C22—H22	120.3
B9—C2—H2	118.2	B30—C22—H22	118.5
B8—C2—H2	118.0	B31—C22—H22	118.9
B6—C2—H2	117.0	B26—C22—H22	117.5
B3—C2—H2	117.3	B23—C22—H22	117.8
N1—C3—C1	120.2 (2)	N2—C23—C21	120.3 (2)
N1—C3—H3A	107.3	N2—C23—H23A	107.3
C1—C3—H3A	107.3	C21—C23—H23A	107.3
N1—C3—H3B	107.3	N2—C23—H23B	107.3
C1—C3—H3B	107.3	C21—C23—H23B	107.3
H3A—C3—H3B	106.9	H23A—C23—H23B	106.9
N1—C4—H4A	109.5	N2—C24—H24A	109.5
N1—C4—H4B	109.5	N2—C24—H24B	109.5
H4A—C4—H4B	109.5	H24A—C24—H24B	109.5
N1—C4—H4C	109.5	N2—C24—H24C	109.5
H4A—C4—H4C	109.5	H24A—C24—H24C	109.5
H4B—C4—H4C	109.5	H24B—C24—H24C	109.5
N1—C5—H5A	109.5	N2—C25—H25A	109.5
N1—C5—H5B	109.5	N2—C25—H25B	109.5
H5A—C5—H5B	109.5	H25A—C25—H25B	109.5
N1—C5—H5C	109.5	N2—C25—H25C	109.5
H5A—C5—H5C	109.5	H25A—C25—H25C	109.5
H5B—C5—H5C	109.5	H25B—C25—H25C	109.5
N1—C6—H6A	109.5	N2—C26—H26A	109.5
N1—C6—H6B	109.5	N2—C26—H26B	109.5
H6A—C6—H6B	109.5	H26A—C26—H26B	109.5
N1—C6—H6C	109.5	N2—C26—H26C	109.5
H6A—C6—H6C	109.5	H26A—C26—H26C	109.5
H6B—C6—H6C	109.5	H26B—C26—H26C	109.5

### Hydrogen-bond geometry (Å, °)

**Table:**

*D*—H···*A*	*D*—H	H···*A*	*D*···*A*	*D*—H···*A*
C2—H2···I1	1.10	3.03	3.946 (3)	141.
C3—H3B···I1	0.97	2.94	3.904 (3)	172.
C23—H23B···I1	0.97	2.96	3.921 (3)	170.
